# Meso-Macroporous Hydroxyapatite Powders Synthesized in Polyvinyl Alcohol or Polyvinylpyrrolidone Media

**DOI:** 10.3390/nano14161338

**Published:** 2024-08-12

**Authors:** Olga S. Antonova, Margarita A. Goldberg, Alexander S. Fomin, Kirill A. Kucheryaev, Anatoliy A. Konovalov, Margarita A. Sadovnikova, Fadis F. Murzakhanov, Aleksey I. Sitnikov, Alexander V. Leonov, Nadezhda A. Andreeva, Dinara R. Khayrutdinova, Marat R. Gafurov, Sergey M. Barinov, Vladimir S. Komlev

**Affiliations:** 1 Baikov Institute of Metallurgy and Materials Science, Russian Academy of Sciences, Moscow 119334, Russia; oantonova@imet.ac.ru (O.S.A.); afomin@imet.ac.ru (A.S.F.); ak357@rambler.ru (A.A.K.); alexei.sitnikov@gmail.com (A.I.S.); andreeva150388@mail.ru (N.A.A.); dvdr@list.ru (D.R.K.); barinov_s@mail.ru (S.M.B.); 2 Department of Functional Nanosystems and High-Temperature Materials, National University of Science and Technology “MISIS”, Moscow 119049, Russia; k_kucheryaev@mail.ru; 3 Institute of Physics, Kazan Federal University, 18 Kremlevskaya Str., Kazan 420008, Russia; margaritaasadov@gmail.com (M.A.S.); murzakhanov.fadis@yandex.ru (F.F.M.); marat.gafurov@kpfu.ru (M.R.G.); 4 Department of Chemistry, Lomonosov Moscow State University, Moscow 119991, Russia; avlenov49@gmail.com

**Keywords:** hydroxyapatite, polyvinyl alcohol, polyvinylpyrrolidone, precipitation synthesis, meso-macroporosity

## Abstract

Mesoporous hydroxyapatite (HA) is widely used in various applications, such as the biomedical field, as a catalytic, as a sensor, and many others. The aim of this work was to obtain HA powders by means of chemical precipitation in a medium containing a polymer—polyvinyl alcohol or polyvinylpyrrolidone (PVP)—with concentrations ranging from 0 to 10%. The HA powders were characterized by X-ray diffraction, Fourier transform infrared spectroscopy, atomic emission spectroscopy with inductively coupled plasma, electron paramagnetic resonance, scanning electron microscopy (SEM), and transmission electron microscopy (TEM). The specific surface area (SSA), pore volume, and pore size distributions were determined by low-temperature nitrogen adsorption measurements, and the zeta potential was established. The formation of macropores in powder agglomerates was determined using SEM and TEM. The synthesis in 10% PVP increased the SSA from 101.3 to 158.0 m^2^/g, while the ripening for 7 days led to an increase from 112.3 to 195.8 m^2^/g, with the total pore volume rising from 0.37 to 0.71 cm^3^/g. These materials could be classified as meso-macroporous HA. Such materials can serve as the basis for various applications requiring improved textural properties and may lay the foundation for the creation of bulk 3D materials using a technique that allows for the preservation of their unique pore structure.

## 1. Introduction

The problem of bone tissue damage caused by various accidents remains topical today. The fact that bone is a mineral–polymer composite has resulted in the development of mineral–polymer composites intended for bone defect repair [1]. Hydroxyapatite (HA, Ca_10_(PO_4_)_6_(OH)_2_) remains the main mineral component of such composites owing to the similarity of its composition to that of the mineral component of bone [2]. The range of polymers applied in this regard is much wider. It should be noted that the introduction of polymers during the synthesis of HA can significantly change its properties, such as the dispersity and morphology [3,4]. The presence of polymers significantly affects the stages of nucleation and crystal growth [5,6]. Thus, in [6], it was shown that the ionic polymer poly(acrylic acid) helps to accelerate the nucleation of HA. Also, Kumar et al. compared cetyltrimethylammonium bromide (CTAB), polyvinylpyrrolidone (PVP), and ascorbic acid as synthesis media and showed that they have significant effects on the morphology and size of HA synthesized by the hydrothermal method. They found that ascorbic acid, CTAB, and PVP induced oriented growth of HA along the c-axis, forming needle-like, rod-like, and fibrous HA, respectively [7].

In recent years, HA has been regarded as a material suitable for applications beyond biomedical ones. Its uses as a sorbent [8,9] or as a support for catalysts [10,11] and several others [12] are being evaluated. In this regard, the importance of controlling the dispersion, morphology, mesoporosity, and pore volume [13] of the resulting materials is increasing. Porosity and pore size are two of the most important characteristics of materials and have a decisive influence on phenomena such as biological behavior (interaction with cells and wettability by biological fluids, i.e., to allow cell migration, proliferation, and differentiation and nutrient–waste exchange), sorption of various pollutants, and adhesion properties (support–catalyst interaction). One of the main applications of mesoporous HA in medicine is as a bone filler (for small defects) and/or as a drug delivery system. The large specific surface area (SSA) (around 100 m^2^/g) makes the material metabolically active and more reactive, more resorbable, and with greater bioactivity [14]. Moreover, the presence of mesopores and a large pore volume allow for the delivery of a significant amount of drugs and also adjustment of their release rates. Thus, in the work in [15], HA was obtained with an SSA of 85 m^2^/g, a pore volume of 0.423 cm^3^/g, and an average pore size of 20 nm, which showed good prospects in vitro for the delivery of ibuprofen.

A number of authors have studied the features of the synthesis of HA in the presence of polymers [16,17]. The most popular are gelatin [18], chitosan [19], and collagen [20]. The interaction between HA crystals and a polyvinyl alcohol (PVA) or polyvinylpyrrolidone (PVP) organic matrix is mainly mediated by hydrogen bonds. PVP and PVA have polyvinyl skeletons with polar groups. The usefulness of PVP and PVA in this field may be associated with a combination of the spatial effect and electrostatic and hydrogen bond effects [21]. It is known that PVA is characterized by a good capability to be crosslinked, both chemically and physically, by a freeze–thaw procedure [22]. PVP is considered a dispersant and a stabilizer, which prevents particle aggregation via the repulsive forces that arise from its hydrophobic carbon chains that extend into solvents and interact with each other (a steric hindrance effect) [23]. The feasibility of obtaining mesoporous HA with SSA of 41.3–63.7 m^2^/g in the presence of PVA was demonstrated by R. Hussain et al.: the powders were synthesized by the wet method using Ca(OH)_2_ as a calcium source [24]. In that work, hydrothermal synthesis in the presence of PVA resulted in a decrease in the particle size of HA and SSA enlargement from 89.47 to 107.40 m^2^/g. On the other hand, synthesis using the precipitation method with subsequent ripening in a mother liquor can help to obtain anion- and cation-substituted mesoporous HA with extremely large SSAs of up to 200 m^2^/g [25,26]. Preparation of pure HA powders with very large SSAs has been a challenge to date.

The polymers PVA and PVP are often used to obtain powdered HA using the sol–gel method [27] and hydrothermal [28] methods. Thus, in the work in [29], HA nanofibers were synthesized by electrospinning in the presence of PVA or PVP, and a comparison of the obtained materials was made. However, there are not enough works dedicated to synthesis using the wet method in the presence of these polymers.

The aim of this work was to compare and find approaches to obtaining meso-macroporous HA powders using a wet chemical precipitation technique with polymers of different types. For instance, PVA ensures the formation of an emulsion in the reactor, thereby likely leading to the emergence of additional interface surfaces; in addition, the emulsion droplets themselves are separate reactors. The introduction of PVP should not cause the formation of an emulsion; the deposition follows the classic sol–gel mechanism, but if a polymer template is present, it could interact both with calcium ions in solution and with particles of newly formed HA, thus affecting crystallization processes. The created materials were compared with those obtained under the same conditions without polymers.

We synthesized mesoporous HA powders by precipitation from aqueous solutions in the presence of either PVA or PVP at a concentration up to 10% and investigated the influence of ripening in the mother liquid for 7 days.

## 2. Materials and Methods

### 2.1. Powder Synthesis

HA powders were prepared by chemical precipitation from aqueous solutions of Ca(NO_3_)_2_·4H_2_O (analytical grade, Labtech Ltd., Moscow, Russia) and (NH_4_)_2_HPO_4_ (analytical grade, Himcity Ltd., Saint Petersburg, Russia). pH values were maintained in the range of 9–11 via addition of a 25% aqueous solution of NH_4_OH (extra-pure grade, Sigma Tek Ltd., Khimki, Russia). The synthesis was carried out in accordance with the reaction:

10Ca(NO_3_)_2_ + 6(NH_4_)_2_HPO_4_ + 8NH_4_OH → Ca_10_(PO_4_)_6_(OH)_2_ + 20NH_4_NO_3_ + 6H_2_O. (1)

During the synthesis from polymer-free solutions, a solution of calcium nitrate was prepared in 200 mL of water (with a Ca ion concentration of 0.521 mol/L) and a solution of ammonium phosphate was prepared in 100 mL of water (with a P ion concentration of 0.624 mol/L). The pH of the calcium nitrate solution was adjusted to 10–11 with NH_4_OH. Then, the ammonium phosphate solution was added dropwise to the calcium nitrate solution with constant stirring. The pH level was monitored using a Testo-206 pH meter and maintained in the range of 10–11.

**Figure 1:**
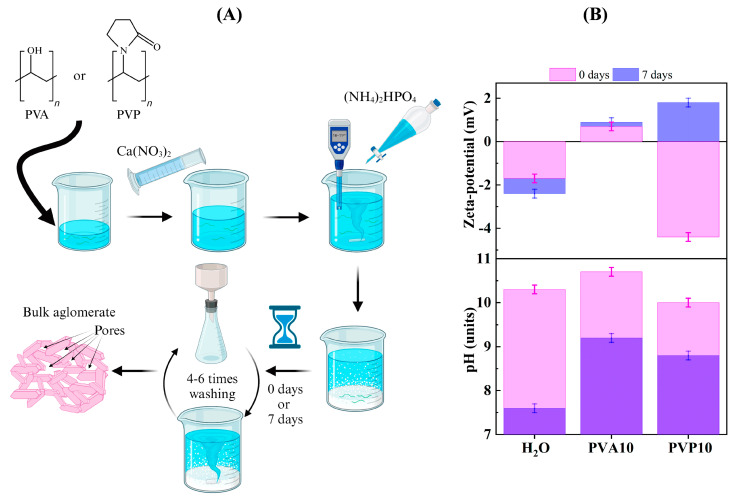
(**A**): The scheme of material processing, (**B**): zeta-potential and pH evolution.

For the synthesis in a polymer medium (Figure 1A), a solution of either PVA (grade 16/1, in accordance with the Russian Federation State Standard GOST 10779-78 [30], Paritet Ltd., Moscow, Russia), or PVP (M_w_ = 10 kDa, grade K-17, Garmoniya Ltd., Saint Petersburg, Russia ) was first prepared with a concentration twice as high as that required for the synthesis of HA (considering that after the addition of the solutions containing calcium and phosphorus, the concentration would decrease by a factor of two). To this end, the polymer powder was introduced in small portions into 150 mL of water with constant stirring on a magnetic stirrer heated at 80 °C; the next portion was added after the previous one had dissolved completely. Calcium nitrate was dissolved in 100 mL of water (calcium ion concentration was 1.041 mol/L), and ammonium phosphate was dissolved in 50 mL of water (phosphorus concentration was 1.248 mol/L). The calcium nitrate solution was then added to the polymer solution and homogenized with an IKA overhead stirrer, after which the pH of the calcium-containing solution was adjusted to 10–11 by means of NH_4_OH. The next step was introduction of a solution of ammonium phosphate dropwise with constant stirring. The pH level was maintained in the range of 10–11.

The resulting suspensions continued to be stirred for 1 h, and after that, they were divided into two equal parts. One part was subjected to washing and filtering immediately (this time point was designated as 0 days of ripening), and the other one was ripened for 7 days at 37 °C. After the completion of the synthesis (including ripening of one part of the suspension), white precipitates were washed with deionized water four to six times to achieve a calculated polymer concentration in the solution of less than 0.01% or neutral pH in the case of synthesis in the polymer-free aqueous medium. The resultant washed precipitate was mixed with ethanol and filtered via a vacuum pump and dried at 60 °C for 24 h. The dried powder was passed through a 65 μm sieve. Concentrations of 2.5, 5.0, and 10.0% of PVA and PVP were chosen in this study (Table 1).

### 2.2. Materials’ Characterization

#### 2.2.1. Suspension Characterization

Properties of HA powders in mother liquor suspensions were studied by zeta potential measurements using electroacoustic spectroscopy with a DT-1200 spectrometer (Dispersion Technology Inc., Bedford Hills, NY, USA). The zeta potential of HA agglomerates was measured directly in the mother liquor prior to filtration and drying of the powders to evaluate the influence of ripening processes. For this purpose, the calibration of the instrument was performed with each mother liquor as a control liquid. In the PVA10-0 suspension, the formation of an emulsion based on polymer gel particles was implemented in addition to the formation of the inorganic precipitate after the synthesis. For calibration, these gel particles were filtered through a mesh with a pore size of 300 µm, and the remaining suspension was filtered with a vacuum pump to obtain a homogeneous mother liquor. The resulting filtrates were used to measure viscosity on a Brookfield LVT viscometer (Brookfield Engineering, Middleboro, MA, USA) (Table 2) and then to calibrate the DT-1200 spectrometer unit individually for each composition of samples. The DT-1200 software was employed to calculate the average particle size with the help of the attenuation coefficient of ultrasonic vibrations of different frequencies on the solid-phase particles. The electrokinetic potential of the sludge suspension was computed from the “colloid oscillation current” measured by the plant sensor. The size of agglomerates in the suspension was analyzed using a Carl Zeiss Axio Observer 3 optical microscope.

#### 2.2.2. Powders’ Characterization

To study the chemical composition of the obtained powders, they were calcined at 900 °C for 2 h and analyzed by AES-ICP (Plasma 3500, NCS Testing Technology Co., Ltd., Beijing, China). All the findings are presented in Table 3.

To study the completeness of the precipitation reaction after the ripening procedure, mother liquor aliquots were taken and investigated by atomic emission spectrometry with inductively coupled plasma (AES-ICP) (Vista Pro, Varian, Palo Alto, CA, USA). Wavelengths of 393 and 366 nm for calcium quantification and 214 and 914 nm for phosphorous quantification were utilized.

Because we calculated the concentrations of calcium and phosphorus ions relative to the entire volume of the synthesis mixture (347 and 208 mmol/L, respectively), and because, from the AES-ICP data, we determined the concentrations of calcium and phosphorus ions in a mother liquor (C_Ca1_ and C_P1_), we could calculate how much calcium and phosphorus were incorporated into the precipitate (C_Ca2_ and C_P2_) according to the equation:

C_Ca2_ = 347 − C_Ca1_ or C_P2_ = 208 − C_P1_ (2)

where C_Ca1_ and C_P1_ are the calcium and phosphorus ions’ concentrations in the mother liquor, and C_Ca2_ and C_P2_ are the calcium and phosphorus ions’ concentrations in the precipitate. All obtained data are presented in Table 4.

The powder materials were characterized by X-ray diffraction (XRD) (Shimadzu XRD-6000, Kyoto, Japan, CuK_α_ radiation, 10–60° [2θ]) with identification of phase composition in the ICDD PDF2 database. Indexing of the peaks was carried out using card ICDD No. 09-0432 (hydroxyapatite).

The Gaussian fit obtained in the Origin software (OriginPro Version 2018 SR1 b9.5.1.195) was used for establishing the peak (002) full width at half maximum (FWHM) to calculate the crystallite size (D) via Scherrer’s equation:

D = kλ/(B_1/2_cosθ), (3)

where k is the shape factor equal to 0.9, λ (0.15406 nm) is the wavelength of X-rays, B_1/2_ is the FWHM of an X-ray reflection in radians, and θ is Bragg’s diffraction angle.

Fourier transform infrared (FTIR) absorption spectra of the samples were recorded using the KBr method on a Nicolet Avatar 330 FTIR spectrometer (Thermo Fisher Scientific, Waltham, MA, USA), and the spectra were obtained in the range from 4000 to 400 cm^−1^ to evaluate functional groups in the samples.

The electron paramagnetic resonance (EPR) method was implemented on a Bruker Elexsys E580 spectrometer with an operating frequency of 9.6 GHz. Transverse spin relaxation (*T*_2_) curves were obtained from room-temperature (297 K) EPR spectra using the Hahn pulse sequence π/2–τ–π–τ–*ESE* (election spin echo), where π/2 = 16 ns, π = 32 ns, and τ = 200 ns (a changing value for *T*_2_, 200 ns to 30 μs) is a time interval between pulses. In pulse mode, the resonance absorption signal was recorded as a dependence of ESE integral intensity on the continuously sweeping external magnetic field *B*_0_. Spin–lattice relaxation curves (*T*_1_) were constructed using the “inversion-recovery” pulse sequence π–*T*–π/2–τ–π–τ–*ESE*, where *T* varied from 0.5 to 300 μs. To generate stable paramagnetic centers within a pure material, the synthesized powders were exposed to X-ray irradiation from a URS-55 source (55 kV, 16 mA, W anticathode) at room temperature for 30 min, resulting in an estimated dose of 10 kGy.

SSA was determined by the Brunauer–Emmett–Teller (BET) method, and pore volume and pore size distributions were assessed using the Barret–Joyner–Halenda (BJH) analyzer model with low-temperature nitrogen adsorption measurements (Micromeritics TriStar Analyzer, Micromeritics Instrument Corporation, Norcross, GA, USA).

From the obtained SSA data, we calculated the average particle size (assuming that the particles had a rounded shape) according to the formula:

D_SSA_ = 6000/(ρ × S), (4)

where D_SSA_ is the particle diameter [nm], ρ is the density of HA [g/cm^3^], and S is SSA [m^2^/g].

Scanning electron microscopy (SEM) was performed on gold-sputtered samples. The SEM analyses were carried out under a Tescan Vega 2 microscope (Tescan, Brno, Czech Republic) in secondary electron (SE) mode. Field emission gun scanning electron microscopy (FEG-SEM) was conducted by means of a FEI Versa 3D instrument. The pore size data were processed using the ImageJ software (Version 1.54g). Particle morphology analyses in the H_2_O-7 and PVP10-7 samples were conducted via transmission electron microscopy (TEM) (JEOL JEM 2100, Tokyo, Japan). Particle size was computed using the ImageJ software via measurement of 100 random particles in each image, followed by statistical analysis. Identification and indexing of selected-area electron diffractions (SAED) were performed with the CrysTBox software (Version 1.10) [31].

## 3. Results

### 3.1. Suspension Properties

These results are summarized in Table 2. For the mother liquors containing polymers, viscosity values were slightly higher as compared to the polymer-free aqueous medium (code-named H_2_O in the Table 1).

During the first 24 h, due to the interaction of PVA with Ca^2+^ and to the high hydroxyl concentration (pH level ≥ 10), the formation of gel particles was detectable at all PVA compositions, resulting in an emulsion.

The ripening in a mother liquor reduced the pH owing to the emergence of HA crystals along with enrichment with bands of chemically bonded OH^−^, as confirmed by subsequent FTIR data (Table 3, Figure 1B). The size of the agglomerates diminished considerably and reached 0.04–0.06 µm when the powders were ripened in polymer-free and PVP solutions. The zeta-potential measurements indicated that the powders had a small double electron layer (DEL) charge. The suspensions showed values that did not go outside ±5 mV. The ripening had no effect on zeta potential of the PVA solution and a negligible effect on the polymer-free aqueous solution. By contrast, the PVP solution manifested an increase in zeta potential from −4.4 to +1.8 mV. The formation of a DEL could be facilitated by Ca^2+^, PO_4_^3−^, OH^−^, CO_3_^2−^, and a partially charged polymer (due to hydroxyl ion dissociation under basic conditions). The results revealed an increase in the contribution of Ca^2+^ ions to the composition of the DEL in the case of PVP. We could also attribute this change to an interaction of the hydrogen of HA hydroxyl groups with the oxygen of the PVP pyrrolidone moiety, resulting in a charge change [32].

According to optical microscopy data (Figure S1), all suspensions contained agglomerates of various sizes, from less than 1 µm to several tens of microns, and the polymer suspension could form a volumetric network that acted as a scaffold for openwork structures of the powders. Furthermore, gel particles were noted in PVA synthesis media.

### 3.2. Chemical Composition of Powders

According to the AES-ICP data (Table 4), all samples obtained immediately after precipitation (ripened for 0 days) showed retention of a considerable amount of calcium ions in solution (with almost complete precipitation of phosphate ions) and manifested a lower Ca/P ratio as compared to stoichiometric HA (Ca/P = 1.67). The synthesis in the presence of any polymer substantially reduced the concentration of calcium ions in the solutions (≤3-fold). Ripening of the precipitate in the mother liquors led to almost complete precipitation of calcium ions and to an increase in Ca/P of up to 1.67. A slight increase in the phosphate ion amount in the mother liquor during the ripening was also observed.

In addition to studying the mother liquors, we investigated the powders using AES-ICP (Table 5). The powder analyses confirmed the increase in the calcium content of the powders after the ripening of precipitates in a mother liquor. We observed a decrease in the concentration of P ions in the powders during the ripening process. Ca/P ratios of the as-synthesized powders showed a similar tendency for an increase as in the case of the calculated Ca/P ratios obtained from the AES-ICP data on the mother liquors. An elevated value compared to the stoichiometric one (1.71 for PVA and 1.83 for PVP) was likely due to partial substitution of phosphate anions in the powders and the incorporation of carbonate anions into the lattice, as confirmed by the FTIR results. The discrepancy between Ca/P in the case of mother liquids and powders analyzed by AES-ICP was negligible and was linked to systematic error and calcium consumption for stitching of the polymer.

### 3.3. XRD Analysis

**Figure 2:**
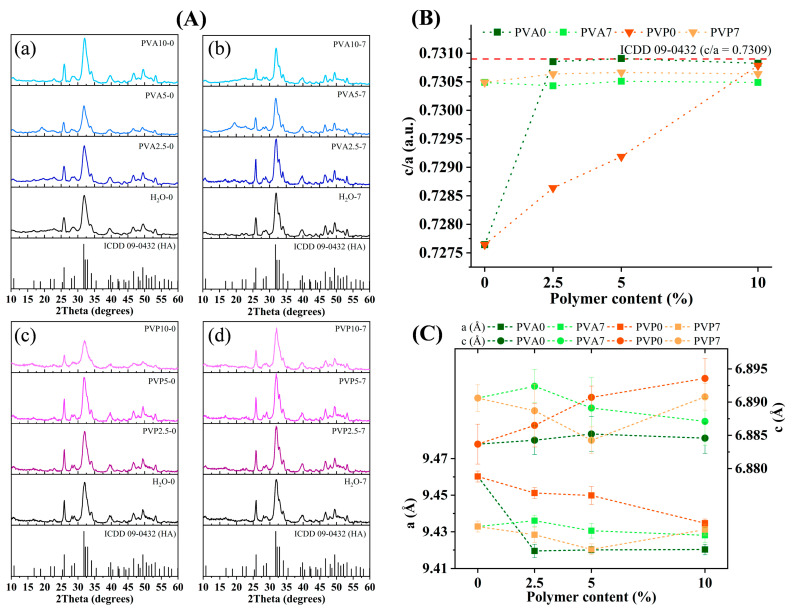
(**A**): XRD data on the powders after the synthesis (**a**,**c**) and ripening in mother liquid for 7 days (**b**,**d**), (**B**): Cell parameters’ ratio (the red dash line corresponds to the *c/a* ratio of the ICDD 09-0432 card) and (**C**): cell parameters’ values.

According to this analysis, all powders had a single phase (hydroxyapatite; ICDD No: 09-0432). After the synthesis (0 days of the ripening), all materials featured broad peaks in the apatite-specific region of the spectrum (Figure 2A(a,c)). The use of PVP resulted in a decrease in the peak intensity and resolution with increasing concentration. After the synthesis in the PVA medium, a decrease in intensity and lower resolution of the peaks were also observed with increasing polymer concentration up to 5%. Nonetheless, a further increase in the concentration to 10% enhanced the peaks’ intensity. When powders were ripened for 7 days, there was an increase in crystallinity in all types of samples. The increase in PVP concentration diminished the crystallinity of the ripened materials similarly to as-synthesized powders. The introduction of PVA initially caused minor growth in the peak intensity at the concentration of 2.5%, and then the intensity declined at higher PVA concentrations. Crystallite size was enlarged very slightly after the ripening for 7 days; only for PVP10 did this value go up noticeably, from 7 to 13 nm (Table 6). The introduction of polymers led to an appreciable decrease in parameter a, with a negligible influence on parameter *c* (Figure 2B,C). The concentration of PVA did not affect the cell parameters, whereas PVP led to a slight noticeable decrease in parameter *a* from 0.9451 to 0.9435 for PVP2.5-0 and PVP10-0, respectively. The ripening for 7 days gave rise to a structure with a *c/a* ratio close to the theoretical one in the case of synthesis with the polymer-free or PVP medium and slightly diminished this ratio in materials synthesized with PVA (Figure 2B).

### 3.4. FTIR Spectroscopy

**Figure 3:**
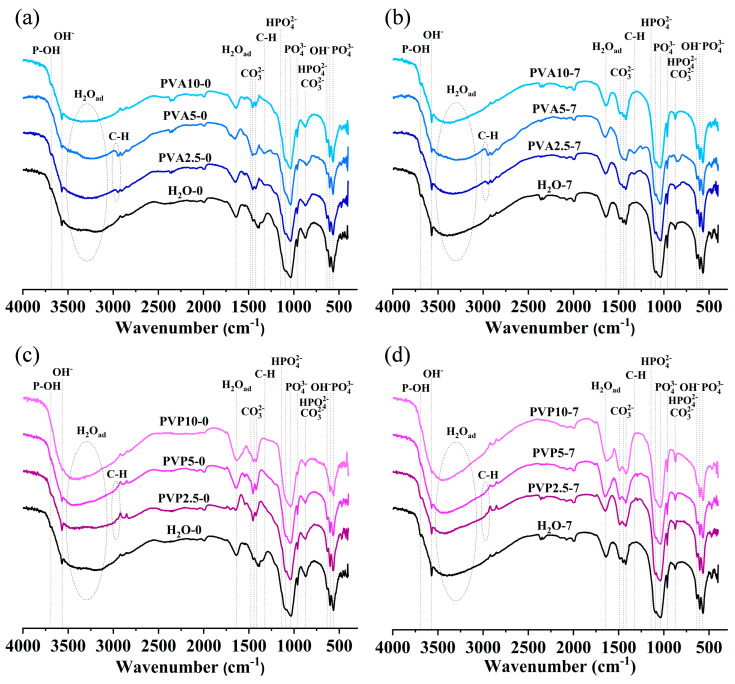
FTIR data on the powders after synthesis (**a**,**c**), and ripening in mother liquid for 7 days (**b**,**d**).

FTIR spectra are presented in Figure 3. All spectra show broad halos from 3500 to 2500 cm^−1^ and peaks at ~1640 cm^−1^ (O-H ν_2_ bending vibration) related to adsorbed water [33]. The presence of hydroxyl group bands at 3570 and 633 cm^−1^ confirms the formation of an HA structure. The introduction of either PVA or PVP substantially weakened the OH bands, except for the PVA10 sample. Several spectral bands, at 3698 or 3737 cm^−1^, are assignable to O-H stretching vibrations of surface P-OH groups [33]. Bands of phosphate groups are less resolved as compared to well-crystallized HA [34]. In the spectrum, asymmetric stretching vibration ν_3_ of PO_4_^3−^ at 1095 and 1043 cm^−1^, ν_1_ PO_4_^3−^ symmetric stretching vibration at 960 cm^−1^, and ν_4_ O-P-O bending vibrations can be distinguished; the latter are characterized by bands at 603 and 568 cm^−1^ [35]. The intensity of ν_1_ PO_4_^3−^ vibrations diminished with increasing PVA and PVP concentrations, except for the PVA10 sample. Ripening considerably enhanced hydroxyl bands and the ν_1_ PO_4_^3−^ band (in the PVA10 sample, the difference was negligible), possibly indicating an improvement of the apatite crystal structure.

There are several bands—at 1558, 1544, 1487, 1465, 1451, 1427, 1412, 1403, 1382, and 873 cm^−1^—and a shoulder at 1507 cm^−1^ that could be attributed to different states of CO_3_^2−^. The bands at 1465, 1412, and 873 cm^−1^ may be related to nonstructural carbonate at the HA surface [36]. The well-pronounced doublets at 1451 and 1427 cm^−1^ with a band at 1412 cm^−1^ were assigned to the B-type of carbonated apatite, whereas the appearance of a band at 1558 and/or 1544 cm^−1^ and the shoulder at 1507 cm^−1^ indicates the formation of A-type carbonated apatite [37]. Thus, predominantly the B-type of carbonated apatite was noted in samples PVA2.5-0 and PVA5-0, whereas PVA10-0 also contained the A-type of carbonated apatite, aside from a band at 1487 cm^−1^ (Figure 3a). Ripening (Figure 3b) resulted in emergence of the 1487 cm^−1^ band in samples PVA2.5-7 and PVA5-7, while in sample PVA10-7, this band lost intensity (see the interpretation below). The presence of bands in the 1550 cm^−1^ region indicates a noticeable introduction of carbonate at the A-position, while most of the carbonate ions were in the B-position. For PVP samples, the presence of both a band at 1544 cm^−1^ and a doublet at 1451 and 1427 cm^−1^ pointed to the formation of the AB-type of carbonated HA (Figure 3c). Ripening of PVP samples (Figure 3d) gave rise to a doublet at 1451 cm^−1^ (whose intensity declined with the growing PVP concentration) and 1421 cm^−1^, which matched the B-type of carbonate substitution, as well as the band at 1487 cm^−1^. This band was noted earlier as atypical and attributed by R. Wilson et al. to the B-type of substitution [38]. Other papers indicate that this vibration corresponds to carbonate ions in amorphous apatite [39], which seems closer to our case.

We also observed bands that can be attributed to the presence of the HPO_4_^2−^ group in the apatite lattice. Namely, there is a shoulder at 1145 cm^−1^ and a broad band at 873 cm^−1^ as a consequence of the superposition of HPO_4_^2−^ and carbonate bands. As stated above, there is a substantial amount of carbonate in the material, both structural and adsorbed, which tends to make it difficult to identify bands attributable to the HPO_4_^2−^ group [40]. Nonetheless, taking into account the variations in elemental composition and the Ca/P ratio (Table 4), it can be said that the HPO_4_^2−^ bands came into being at a substantial deviation from the stoichiometric value of 1.67. The ripening process induced enrichment of the material with calcium. As a consequence, the Ca/P ratio was higher than the stoichiometric one. This alteration led to a noticeable weakening to the point of disappearance of the vibrations that can be attributed to the presence of the HPO_4_^2^-group.

The spectra of samples PVA2.5 and PVA5 contain weak bands near 2940 cm^−1^ and at 1328 cm^−1^, which may be related to C-H [41]. Meanwhile, other C-H bands, at 910 and 831 cm^−1^, which have been pointed out by I.Y. Prosanov et al. [41], are almost undetectable. No additional polymer-related bands were noticed in PVP samples. Thus, these data indicate a high degree of polymer removal because of the washing procedure without heat treatment in this study.

### 3.5. EPR Spectroscopy

**Figure 4:**
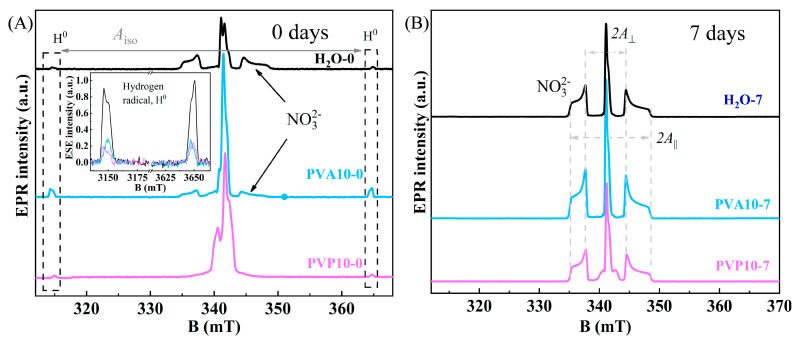
(**A**): EPR spectra of X-ray-irradiated HA samples with various polymers after chemical synthesis, where the inset shows detailed changes of hydrogen radical signals. (**B**): experimental data for samples after ripening for 7 days.

The examined samples of pure HA and powders synthesized in the presence of the polymers, regardless of the ripening time in a mother liquor, did not produce EPR signals. Irradiation of materials by a moderate-dose X-ray source caused the formation of free radicals with a nonzero electron spin. Processing of materials under moderate X-ray irradiation gave rise to free radicals with a nonzero electron spin (*S* > 0). The most common radiation-induced center in HA was the nitrogen radical, which is deservedly recognized as a reliable spin probe for studying a local environment [42]. Figure 4A shows EPR spectra of X-ray-treated samples obtained immediately after chemical synthesis. As expected, pure HA yielded a nitrogen radical signal consisting of three hyperfine structure components. The introduction of polymers during the synthesis induced a major alteration of the EPR spectrum. Specifically, hyperfine splitting components of the NO_3_^2−^ radical decomposed (PVA), whereas in the PVP case, the resonance absorption lines broadened to the extent of complete disappearance. Distortion of the shape and width of the EPR lines by the polymers is undoubtedly associated with deterioration of HA crystallinity. In Figure 4A, one can also see the presence of hydrogen radical H^0^ with isotropic hyperfine splitting *A*_iso_ = 50 mT (1420 MHz) by the contact Fermi interaction mechanism. Comparative analysis in the insets of Figure 4A points to a significant increase (approximately fivefold) in the amount of hydrogen radicals when the PVA polymer was used. The intensity of hydrogen signals in HA-PVP remained at the same level as in pure HA. The PVA-containing samples featured an interaction between hydrophilic OH^−^ groups and calcium ions, and this interaction could have contributed to the increase in H^0^ radical intensity. These phenomena contributed to the decrease in the particles’ growth during the ripening in the mother liquor. From the EPR spectroscopy findings, it can be concluded that the PVA polymer enveloped an HA nanoparticle, thereby clogging the pores along with residual water inside owing to emulsion formation. On the contrary, the PVP polymer filled pores of HA just as in the case of water, thus resulting in the appreciable decrease in particle enlargement (at 7 days).

Figure 4B presents EPR spectra of the same samples after ripening for 7 days. Hyperfine splitting components with *A*_⊥_ = 6.7 mT and *A*_||_ = 13.8 mT, which were due to an anisotropic interaction of the electron spin of the NO_3_^2−^ radical with the ^14^N nucleus (*I* = 1, natural abundance 99.64%), have very narrow lines with high spectroscopic resolution. Spin–spin (T_2_ = 1.7–3.1 μs) and spin–lattice (T_2_ = 25.8–71.4 μs) relaxation times measured in the samples under study are in good agreement with earlier results on single-phase HA [42]. The findings of our EPR analysis are also consistent with XRD data on the formation of highly crystallized porous samples in a short period with the washing out of polymers from the pores.

### 3.6. Textural Properties of the Powders According to Measures of Nitrogen Adsorption

**Figure 5:**
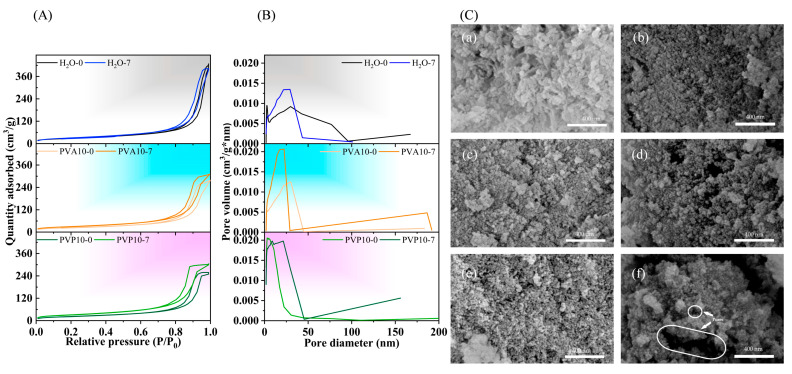
(**A**): Nitrogen adsorption–desorption isotherms of the powders after the synthesis (H_2_O-0, PVA10-0, PVP10-0) and ripening for 7 days (H_2_O-7, PVA10-7, PVP10-7). (**B**): The corresponding BJH pore size distributions of the powders. (**C**): Morphology of HA powders: H_2_O-0 (**a**), H_2_O-7 (**b**), PVA10-0 (**c**), PVA10-7 (**d**), PVP10-0 (**e**), and PVP10-7 (**f**).

Nitrogen sorption isotherms of the powders after the synthesis and ripening for 7 days in polymer-free or PVA- or PVP-containing media are presented in Figure 5A. According to BET method data, the powders’ adsorption–desorption isotherms were of type IV according to IUPAC, thus pointing to the formation of pores with sizes in the range of 2 to 50 nm, characterizing a material as a mesoporous one [43].

Hysteresis loops of materials synthesized in the polymer-free aqueous medium were attributed to H3-type behavior. Loops of this type could be given by nonrigid aggregates of plate-like particles, but also by pore networks consisting of macropores that are not completely filled with a pore condensate [44]. According to the pore size distribution data, there was a broad range of pores with sizes of up to 100 nm. At the same time, the maximum on the distribution curve of pore size was 30.15 nm. The ripening in the polymer-free mother liquor induced transformation into a H2(b) hysteresis loop, which is associated with complex pore structures in which network effects are important. This type of adsorption–desorption behavior is characteristic of a material having a corpuscular structure with an irregular pore shape. Some pores are blocked, but the size distribution of pore neck widths is rather wide. The maximum on the pore distribution curve is located between 21.9 and 29.5 nm. The SSA of H_2_O-0 samples was found to be 101.3 m^2^/g, and in H_2_O-7, its value reached 112.2 m^2^/g (Table 7).

The introduction of PVA or PVP into the synthesis media yielded H2(b)-type hysteresis loops after the synthesis. At the lowest concentration of a polymer (2.5%), after the synthesis, the materials manifested almost parallel positioning of the adsorption and desorption branches, indicating a transition from the H3 type to H2(b) type. For all materials at high relative pressure levels (0.9–1.0 p/p_0_), the isotherms showed an increase, which is typical for macroporous materials [45].

The higher PVA content in the synthesis media induced a shift of the maximum on the pore size distribution curve from 21.9 to 30.0 nm, but a minor amount of a macroporous substance was also observed on the distribution curve. The ripening resulted in a narrowing of the main pore size distribution region, with a maximum at 17.4–17.5 nm regardless of PVA concentration and a tiny rise in the total adsorbed nitrogen volume. The macroporous region also remained. Compared to samples synthesized in the polymer-free aqueous medium, the application of PVA reduced SSA, pore size, and total pore volume.

The synthesis in the PVP medium at a low concentration caused a slight decrease in SSA values of the as-synthesized materials, but the increasing polymer amount resulted in appreciable growth of SSA, up to 158.0 m^2^/g. At the same time, the pore size and total pore volume diminished as compared to materials from the polymer-free medium. For instance, when 10% PVP was applied to the synthesis, the powders featured capillary condensation in mesopores in a wide relative pressure range of 0.5 to 1.0 p/p_0_. This material is characterized by the smallest pore diameters, with a maximum at 3.6 nm, but a macroporous region is absent. PVP2.5-0 and PVP5-0 showed enlargement of the pore size distribution, with peaks at 30.1 and 29.6 nm, and the average pore sizes were 15 and 16 nm, respectively. The ripening caused a dramatic increase (almost twofold) in the total pore volume up to 0.71 cm^3^/g, together with enlargement of SSA up to 195.8 m^2^/g and of the average pore size from 8 to 12 nm for PVP10-7. The obtained total pore volume exceeded 0.6 cm^3^/g, as previously demonstrated for samples synthesized on different hard carbon templates [46]. Likewise, soft templates are characterized by lower values: 0.31–0.44 cm^3^/g for a surfactant-templating system [47]; 0.19 cm^3^/g for a cetyltrimethylammonium bromide template [48]; or 0.45–0.48 cm^3^/g, which is reached with a nonionic surfactant based on Pluronic P123 [49].

The pore sizes in all types of our samples were comparable to those estimated by means of BET particle size, except for PVA5-0/7 powders, which had the smallest SSA.

### 3.7. SEM Investigation

The obtained powders were formed by loose porous agglomerates (Figure 5C). The powder particles precipitated in the polymer-free medium were characterized by rod-like morphology with lengths up to 50 nm and widths of 10–20 nm. It is noteworthy that, in the case of PVA and PVP media, the morphology changed to an almost spherical one, with sizes smaller than 10 nm. Similar morphology has been documented earlier for PVA-synthesized HA obtained by the microemulsion method [50]. The process of ripening in the polymer-containing media led to further growth of the particles’ sphericity and equiaxiality. We could not detect a noticeable change in particle size; the size stayed at the nanoscale level. The macroporosity seen in the as-synthesized materials is characterized by pores up to 55 nm for PVA-derived powders and 70 nm for PVP-derived powders. In as-synthesized materials from the polymer-free medium, macropores were not noticed. The ripening enlarged the quantity and size of the macropores. Estimated values for samples from the polymer-free medium were up to 100 nm; for samples from PVA media, up to 170 nm; and for samples from PVP media, up to 650 nm. The macropores seen in Figure 5C(f) confirmed the BET data. It should be noted that no traces of any polymers were observed during the SEM.

### 3.8. TEM Investigation

**Figure 6:**
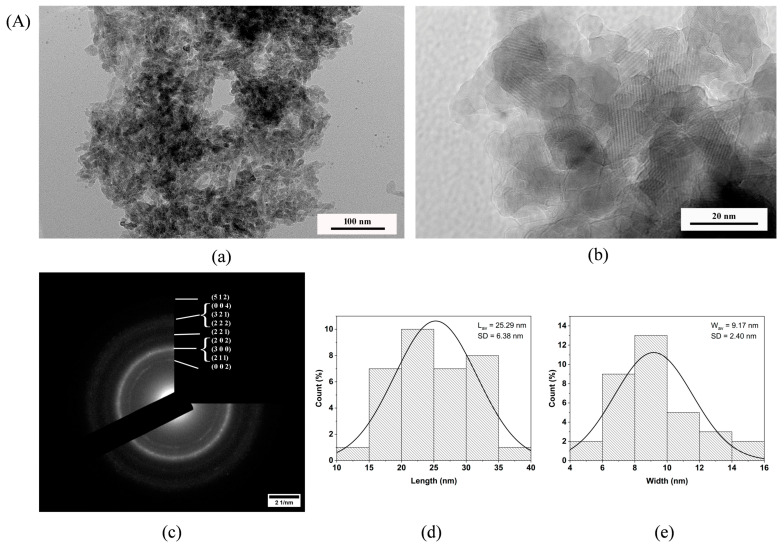

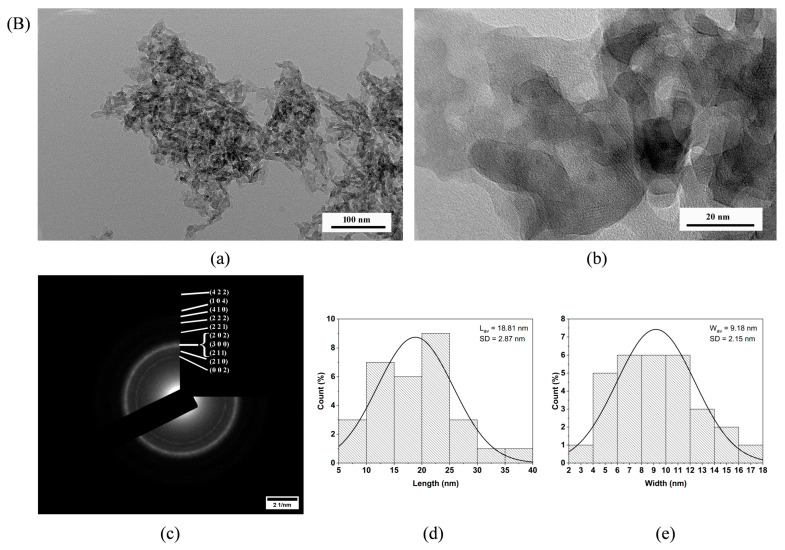
TEM data for (**A**): H_2_O-7 (**a**–**e**); (**B**): PVP10-7 (**a**–**e**). Micrographs (**a**,**b**), SAED (**c**), and particle size distribution (**d**,**e**).

According to the transmission electron microscopy (TEM) data, the powders H_2_O-7 and PVP10-7 (Figure 6) formed particles with plate-like morphology, looking like ellipsoids and rods. The average particle size varied slightly. The average lengths (L) were 25.29 nm for H_2_O-7 and 18.81 nm for PVP10-7, while their average widths (W) were almost the same (9.17 nm for H_2_O-7 and 9.18 nm for PVP10-7). Thus, the aspect ratio of the particles (L/W) showed that ripening in the PVP solution compared to ripening in the polymer-free medium led to a marked difference (2.76 vs. 2.05), with the particles shrinking to a more rounded shape, as was also confirmed by the presence of a certain amount of almost spherical particles in the case of the PVP medium.

In both powders, the particles assembled into rather loose agglomerates. In the H_2_O-7 sample, however, denser packing of the particles in agglomerates was observed. In the case of PVP10-7, the agglomerates were looser, and it was noted that the particles formed openwork structures during the packing process.

The selected-area electron diffraction (SAED) data (Figure 6(Ac,Bc)) confirmed the results of the XRD analyses, indicating the emergence of the pure HA phase in all the tested samples. The crystallinity of powders obtained in the polymer-free medium was somewhat higher than that in powders obtained in a PVP medium, as evidenced by single-point reflections in the electron diffraction pattern of the sample H_2_O-7. These findings are consistent with the XRD data on the lower crystallinity of the PVP10-7 powder compared to H_2_O-7. The decrease in crystallinity was associated with a more intensive nucleation process during precipitation in polymer-containing media, with further simultaneous dissolution and recrystallisation processes during ripening, and with the formation of smaller particles.

## 4. Discussion

In the past decade, mesoporous HA materials have aroused much interest because of their biomedical applications, e.g., as bone fillers, sensors, and drug carriers [51,52], as well as environmental applications, including the adsorption of soil contaminants [53] and of water contaminants [54], gas sensor applications [55], catalysts [56,57,58], and support systems for catalysis [59,60,61] (Graphical Abstract A). All these areas require controlled textural properties, including high porosity, large surface area, and three-dimensional ordered passageways [13]. At the same time, the transition to meso-macroporous materials is opening up new frontiers in materials science. The reason is the possibility of incorporating macropores into mesoporous materials and the consequent combined advantages of mesoporous and macroporous structures [62]. Meso-macroporous materials have been constructed from silica-based substances [63,64], metal oxides [65], aluminum [62], titanium [66], zirconium phosphates [67], and glasses in SiO_2_-P_2_O_5_ [68] or SiO_2_-CaO-P_2_O_5_ [69] systems, and are suitable for a wide range of applications from catalysis and separation to electrochromic devices, fuel cells, and bioactive materials. To the best of our knowledge, data on meso-macroporous calcium phosphates including HA have not been published before.

The synthesis of meso-macroporous HA in the presence of PVA or PVP could be regarded as synthesis on a soft template [13]. Templates are employed to control the nucleation and growth of HA powder particles and, therefore, to adjust their size, size distribution, morphology, pore structure, pore size, and pore size distribution. The main distinction of our work is the use of ripening without additional thermal treatment. A heat treatment could enlarge the powder particle size and prevent the formation of hollow cavities or mesopores between the particles. We removed the polymers by means of washing with distilled water, and this procedure did not alter the morphology of the powders after the synthesis. In the process of HA powder ripening in a mother liquor, non-Ostwald behavior may be achieved when an improvement of textural properties linked with recrystallization of particles can be obtained along with the formation of more spherical particles and a decrease in the agglomerates’ density [70]. It is noteworthy that effects of the polymer were different in our work (Graphical Abstract B). PVA-containing samples showed the formation of an emulsion with prevention of a sol based on the mother liquor and HA particles. At the same time, their viscosity was higher as compared to both polymer-free and PVP media, indicating retention of some PVA in the mother liquor.

In the case of PVP, the viscosity of the solution did not increase much, even though its molecule contains a hydrophilic component of the pyrrolidone moiety. The concentrations of Ca^2+^ and PO_4_^3−^ remaining in the mother liquor were nearly two times higher as compared to the polymer-free and PVA media (Table 4). We could theorize that PVP acts as a capping agent [21] and fills pores of HA, resulting in a deficit of the additional calcium and phosphorous source on the particles’ nucleus surfaces and preventing particle size enlargement during ripening. Only the PVP solution underwent a noticeable zeta-potential change, which included a change from the negative to positive sign of the charge. This switch of the zeta-potential charge can be ascribed to enrichment with Ca^2+^ ions on the DEL during the ripening and the reaction of the pyrrolidone moiety of PVP with hydroxyl groups of HA, according to the mechanism proposed in ref. [32]. We observed a change in pH from 10.0 to 8.8 during the ripening of PVP samples. In refs. [71,72], a change in the sign of the zeta-potential charge was also documented for systems containing PVP when the pH of the medium was altered. This could indicate adsorption of the ions on the surface of HA, aside from the recrystallization of the HA lattice during the ripening.

In our work, as a result of the synthesis of powders by chemical coprecipitation in the presence of PVA or PVP, all the obtained powders bore a resemblance to the structure of precipitated apatite regardless of the concentration and nature of the polymer. The introduction of a polymer reduced crystallinity relative to the HA obtained in the polymer-free aqueous medium. The higher polymer concentrations had a more pronounced effect. These data confirm the results published in ref. [7] about HA synthesized by the hydrothermal method in the presence of PVP. Simultaneously, the ripening in polymer-free, PVA, or PVP media for 7 days here lead to higher crystallinity of HA, as in [70]. According to our AES-ICP data on mother liquors and powders, freshly precipitated powders were calcium-deficient HA (Ca/P = 1.52–1.62 and 1.51–1.59 according to solution and powder data, respectively). When PVA or PVP was introduced into the synthesis medium, the concentration of Ca^2+^ ions diminished appreciably (threefold) in solution, with an expected increase in the precipitate regardless of the nature of the polymer. Ripening of the powders for 7 days caused further recrystallization, with a decline of the Ca^2+^ concentration in the mother liquor. As a consequence, for all types of samples, analysis of the solution revealed a Ca/P ratio of 1.67, characteristic of HA. Meanwhile, a slight increase in the phosphorous amount in the PVA- and PVP-containing mother liquors was observed, which could be explained by dissolution during the recrystallization of HA, with partial substitution of PO_4_^3−^ by CO_3_^2−^ that was absorbed by the solution from the air [73]. This process was more pronounced for polymer-containing synthesis media. This finding was confirmed by AES-ICP in the powders; AES-ICP showed the increase in the Ca/P ratio.

According to FTIR spectroscopy data, the ripening of the powders obtained in the aqueous medium contributed to the enhancement of absorption bands of hydroxyl and phosphate groups with simultaneous disappearance of the HPO_4_^2−^ group, meaning the formation of a more stoichiometric apatite structure. A similar pattern was noted in samples obtained in the PVP medium, but this was also accompanied by strengthening of the CO_3_^2−^ bands (ν_2_ doublet at 1451 and 1414 cm^−1^). These bands are characteristic of B-type carbonated HA [74,75,76], i.e., of substitution of phosphate groups. The A-type of substitution (bands near 1550 cm^−1^) was registered too, but its intensity was somewhat lower than that of B-type bands, implying predominance of the B-type substitution in both PVA and PVP media. It should be pointed out that the ripening produced a 1487 cm^−1^ band in the PVP experiments (except for the PVP10-0 sample), which matches carbonate ions in amorphous apatite. In all samples, the presence of unstructured carbonate was documented.

All the obtained powders after the synthesis were characterized by large SSA, which was in the range from 73.7 m^2^/g (PVA5-0) to 158.0 m^2^/g (PVP10-0) and increased by up to 82.5 to 195.8 m^2^/g, respectively, after the ripening. The introduction of polymers into the synthesis on the first day led to a decrease in SSA values in PVA-derived samples and at low concentrations of PVP. According to our D_SSA_ calculation, the initial particle size changed slightly and was in the range of 11–13 nm. Thus, the decrease in D_SSA_ could be attributed to an assembly of denser agglomerates and a difference in the particles’ length/width (L/W) aspect ratio and morphology, as estimated by TEM, similarly to our previous results presented in [61]. On the other hand, the 10% PVP experiments featured a substantial drop in D_SSA_ down to 7 nm after the synthesis. This effect was related to the assembly of a PVP network in the mother liquor, resulting in more pronounced HA nucleation. The recrystallization processes during the ripening contributed to an increase in SSA in all our experiments.

We also consider PVP a dispersant because we observed ordered elongated distances between particles in the TEM images. Earlier, a uniform spherical multistructure crystal cluster of HA was obtained on a PVP template combined with calcium citrate chelation; the resultant crystal cluster was ~1 µm in size, and single sheets of HA were 200–300 nm in length and 30–50 nm in width owing to synergy between PVP and calcium citrate chelation [47]. Elsewhere, rodlike crystals with lengths up to 100 nm and an L/W aspect ratio of 5 were prepared by Y. Zhang and J. Lu [21] via mild biomimetic synthesis in the presence of HNO_3_ and H_3_PO_4_ at very low concentrations of Ca^2+^ and PO_4_^3−^ ions and PVP (its concentration did not exceed 0.28 mmol/L); morphological alterations linked with Ostwald behavior were observed at 40 and 60 °C after 5 days of ripening. On the other hand, data on textural properties are not presented in that paper. In our work, we attained SSA of 158.0 m^2^/g after the synthesis, and it enlarged up to 195.8 m^2^/g after the ripening, thereby indicating non-Ostwald behavior of the materials. It is important to note that the resultant samples possessed very large pore volumes of up to 0.71 cm^3^/g, which is comparable with organosilica [77] and carbon-based materials [78].

Our analysis of the hysteresis loops of the powders and examination of pore size distributions pointed to the presence of not only mesoporosity, but also macroporosity in the powders. Isotherms of all the materials showed increases under high relative pressures (0.9–1.0 p/p_0_), which is typical for macroporous materials, as previously demonstrated for silica [45,69]. The average pore size of our materials was in the range of 8 to 18 nm or 12 to 16 nm for as-synthesized and ripened samples, respectively. Additionally, via FEG-SEM, we detected pores with sizes up to 170 nm in PVA experiments and up to 650 nm in PVP experiments in ripened powders. The synthesis and ripening in the PVA media showed a slight decrease in textural properties as compared to PVP experiments. We can hypothesize that, during the ripening in PVA media, processes of emulsion formation with subsequent gel sedimentation produced a meso-macroporous structure, albeit with a lower pore size distribution in the mesoporous area and a corresponding decline in SSA and pore volume values. This process should be more deeply investigated in the future.

Our TEM data indicated the formation of loose agglomerates with openwork structures. Thus, in this paper, HA can be classified as a disordered meso-macroporous powder [79], which is being reported for the first time to the best of our knowledge. Meso-macroporous materials have received significant attention in the last few decades and can lead to major advances in emerging applications in catalysis, adsorption research, sensor studies, and biotechnological fields [79,80].

## 5. Conclusions

Meso-macroporous HA powders were synthesized via chemical precipitation in the presence of PVA or PVP with a concentration of up to 10% as a template. The effects of the nature and concentration of the polymer template and of ripening on the formation of powder materials were investigated. The largest SSA and total pore volume were achieved in the powder sample synthesized in the presence of 10% of PVP after ripening for 7 days, which reached 195.8 m^2^/g and 0.71 cm^3^/g, respectively. It is worth mentioning the formation of not only the mesoporous structure of the resulting particles, but also noticeable macroporosity, judging by BET with BJH calculations and FEG-SEM and TEM data. Accordingly, the obtained materials could be classified as meso-macroporous HA, which is reported for the first time to our knowledge. Such materials can serve as the basis for various applications in catalysis, purification research, and biomedicine, for example, for drug-delivery systems or protein carriers, and may lay the foundation for the creation of bulk 3D materials using a technique that allows their unique pore structure to be preserved.

## Figures and Tables

**Table 1:** Names of the samples and reaction conditions.

Sample	Medium	Concentration, %	Ripening Time, Days
H_2_O-0	H_2_O *	-	0
H_2_O-7	H_2_O *	-	7
PVA2.5-0	PVA	2.5	0
PVA2.5-7	PVA	2.5	7
PVA5-0	PVA	5	0
PVA5-7	PVA	5	7
PVA10-0	PVA	10	0
PVA10-7	PVA	10	7
PVP2.5-0	PVP	2.5	0
PVP2.5-7	PVP	2.5	7
PVP5-0	PVP	5	0
PVP5-7	PVP	5	7
PVP10-0	PVP	10	0
PVP10-7	PVP	10	7

* H_2_O: a polymer-free aqueous medium.

**Table 2:** Dynamic viscosity of mother liquors (max concentration).

Mother Liquor	Concentration, %	Viscosity, mPa·s
H_2_O	-	1.0
PVA	10	1.5
PVP	10	2.5

**Table 3:** pH values, agglomerate size, and zeta potential (measurements were repeated 3 times).

Sample	pH Values		Agglomerate Size, μm *		Zeta potential, mV **	
	0 Days	7 Days	0 Days	7 Days	0 Days	7 Days
H_2_O	10.3 ± 0.02	7.5 ± 0.01	0.18	0.04	−1.7	−2.4
PVA10	10.7 ± 0.03	9.2 ± 0.04	0.11	0.10	+0.7	+0.9
PVP10	10.0 ± 0.02	8.8 ± 0.02	0.23	0.06	−4.4	+1.8

* Standard deviation: ±0.05 μm. ** Standard deviation: ±0.2 mV.

**Table 4:** Calculated chemical composition of mother liquors and powders (AES-ICP).

Sample	C_Ca1_ *, mmol/L	C_P1_ *, mmol/L	C_Ca2_ **, mmol/L	C_P2_ **, mmol/L	Ca/P
H_2_O-0	31.00	0.01 (1)	316.00	207.99	1.52
H_2_O-7	0.56	0.01 (5)	346.44	207.98	1.67
PVA10-0	12.43	0.01 (8)	334.57	207.98	1.61
PVA10-7	0.64	0.17 (6)	346.36	207.82	1.67
PVP10-0	9.72	0.08 (6)	337.28	207.91	1.62
PVP10-7	1.07	0.27 (7)	345.93	207.72	1.67

* C_Ca1_ and C_P1_ are calcium and phosphorus ions’ concentrations in a mother liquor; ** C_Ca2_ and C_P2_ are calcium and phosphorus ions’ concentrations in a precipitate.

**Table 5:** Chemical composition of powders (AES-ICP data).

Sample	Ca Concentration *	P Concentration *	Ca/P Molar Ratio
	wt.%	wt.%	
H_2_O-0	36.2	18.6	1.51
H_2_O-7	36.8	17.7	1.61
PVA10-0	36.3	17.7	1.59
PVA10-7	37.9	17.2	1.71
PVP10-0	36.5	17.8	1.59
PVP10-7	38.4	16.3	1.83

* standard deviation: ±0.05%.

**Table 6:** The average crystallite size.

Sample	H_2_O-0/ H_2_O-7	PVA2.5-0/ PVA2.5-7	PVA5-0/ PVA5-7	PVA10-0/ PVA10-7	PVP2.5-0/ PVP2.5-7	PVP5-0/ PVP5-7	PVP10-0/ PVP10-7
D, nm	11 (2)	12 (2)	11 (9)	11 (7)	13 (0)	13 (4)	7 (0)
	12 (8)	14 (4)	12 (5)	14 (4)	15 (1)	14 (1)	12 (7)

**Table 7:** SSA, porosity, and particle size of the powders.

Sample	SSA, m^2^/g	D_SSA_, nm	Total Pore Volume, cm^3^/g	Pore Size, nm
H_2_O-0	101.3	20	0.66	23
H_2_O-7	112.2	18	0.62	19
PVA2.5-0	86.9	23	0.42	18
PVA2.5-7	107.9	19	0.48	16
PVA5-0	73.7	27	0.27	12
PVA5-7	82.5	24	0.29	12
PVA10-0	81.4	25	0.40	16
PVA10-7	113.4	18	0.47	13
PVP2.5-0	88.5	23	0.37	15
PVP2.5-7	139.3	14	0.52	12
PVP5-0	94.8	21	0.42	16
PVP5-7	139.1	14	0.51	12
PVP10-0	158.0	13	0.37	8
PVP10-7	195.8	10	0.71	12

## Data Availability

Data is contained within the article and Supplementary Material.

## Supplementary Materials

The following supporting information can be downloaded at: https://www.mdpi.com/article/10.3390/nano14161338/s1, Figure S1: Micrographs of suspensions of HA in a mother liquor; H_2_O-3 (A), H_2_O-7 (B), PVA2.5-3 (C), PVA2.5-7 (D), PVA5-3 (E), and PVA5-7 (F). Figure S2: Morphology of HA agglomerates; H_2_O-0 (A), H_2_O-7 (B), PVA5-0 (C), PVA5-7 (D), PVA10-0 (E), PVA10-7 (F), PVP5-0 (G), PVP5-7 (H), PVP10-0 (I), and PVP10-7 (J).
