# Supplementary material for: Meso-Macroporous Hydroxyapatite Powders Synthesized in Polyvinyl Alcohol or Polyvinylpyrrolidone Media
Source: Nanomaterials (Basel). 2024 Aug 12;14(16):1338. doi: 10.3390/nano14161338 (PMC11357263; doi:10.3390/nano14161338)
Supplement: Supplementary file 1 [file nanomaterials-14-01338-s001.zip › nanomaterials-3116745-supplementary.pdf]

Supplementary Materials

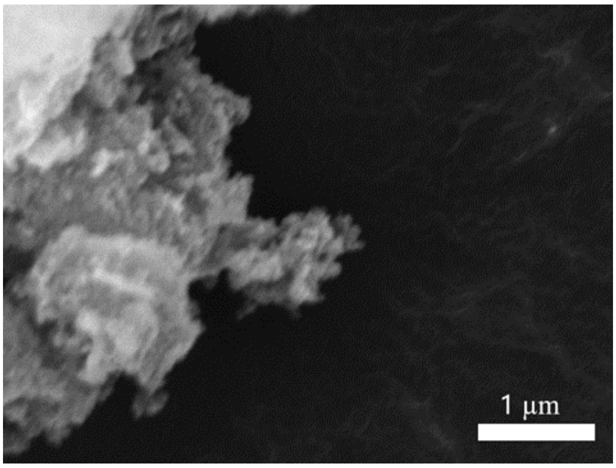

(a)

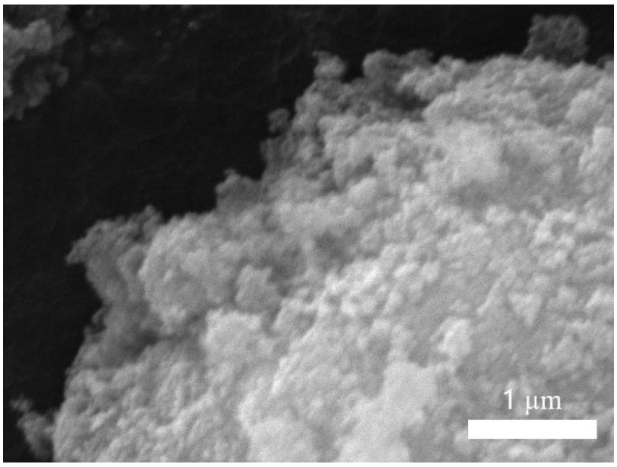

(b)

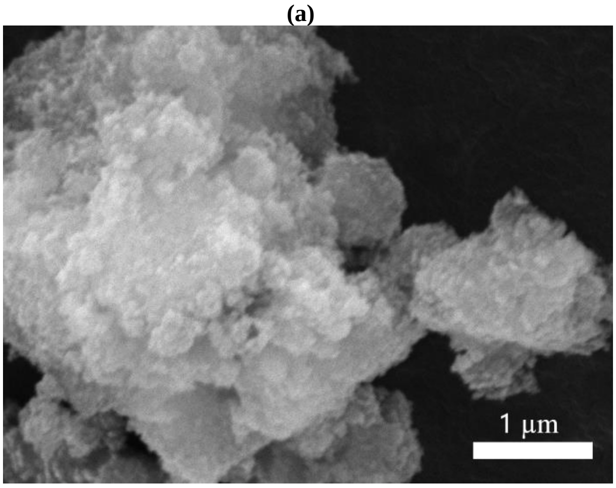

(c)

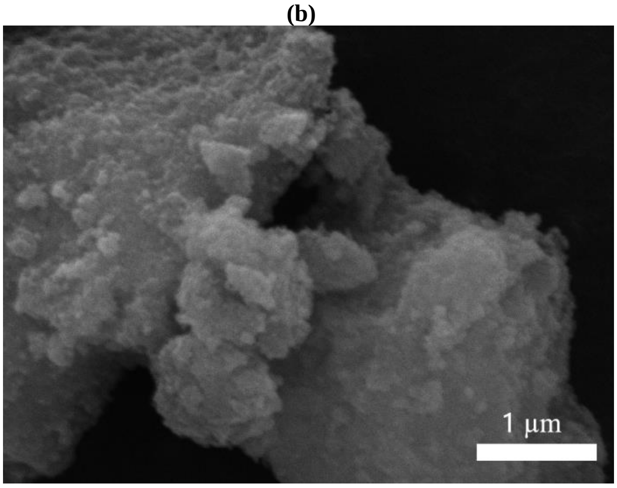

(d)

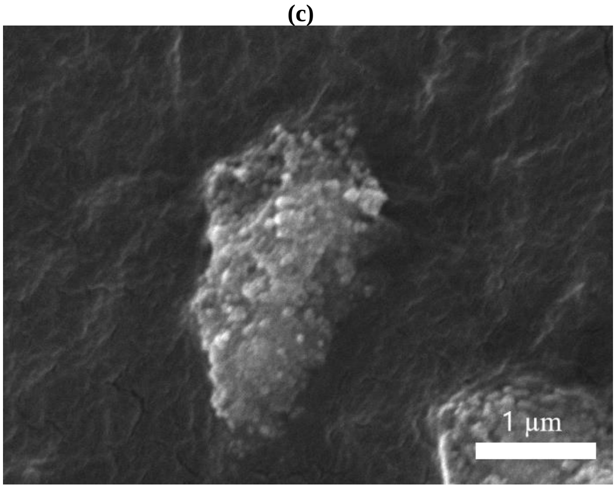

(e)

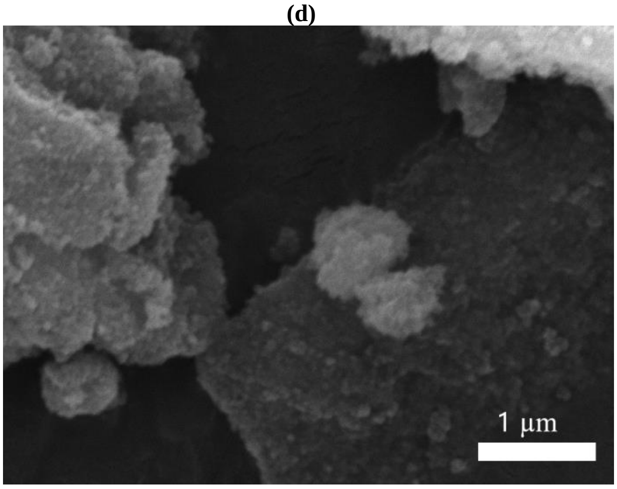

(f)

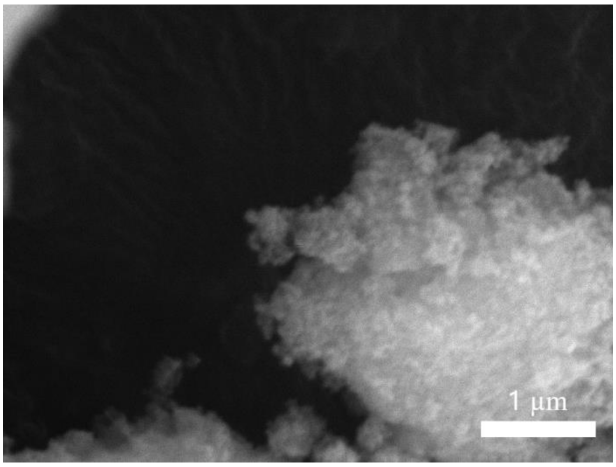

(g)

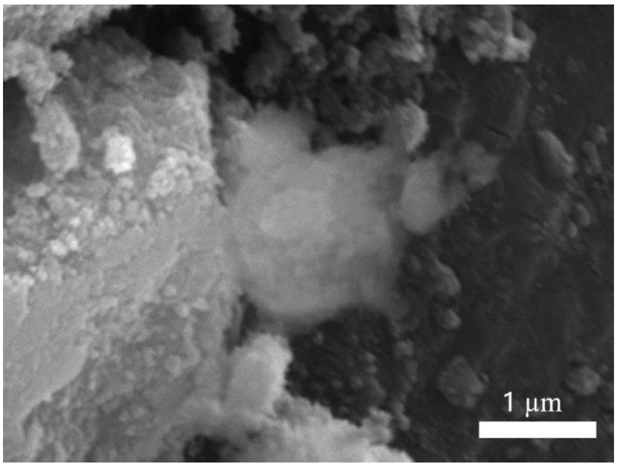

(h)

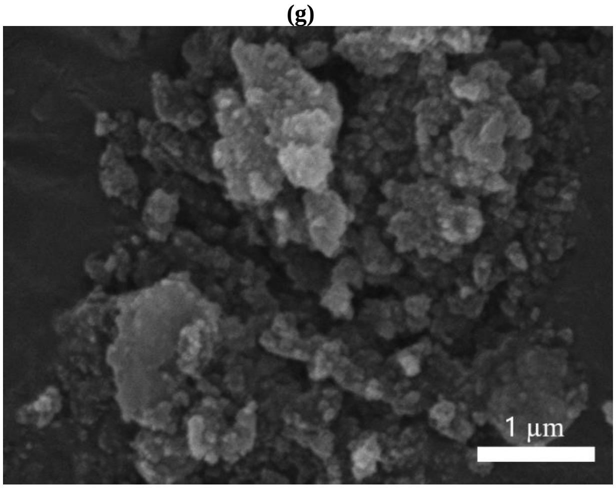

(i)

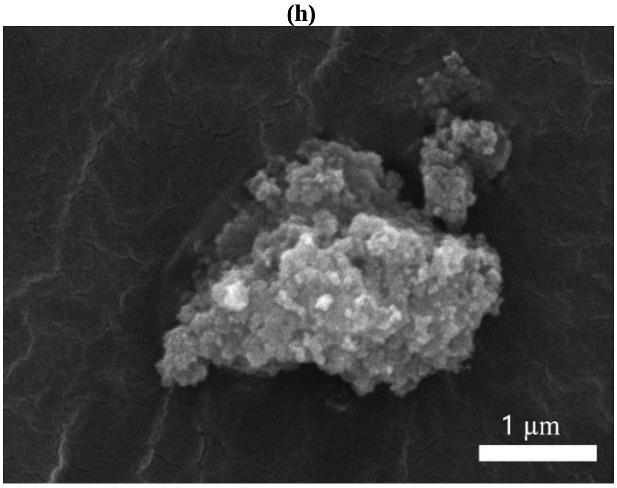

(j)

Figure S1. Morphology of HA agglomerates: H_2O-0 (**a**), H_2O-7 (**b**), PVA5-0 (**c**), PVA5-7 (**d**), PVA10-0 (**e**), PVA10-7 (**f**), PVP5-0 (**g**), PVP5-7 (**h**), PVP10-0 (**i**), and PVP10-7 (**j**).

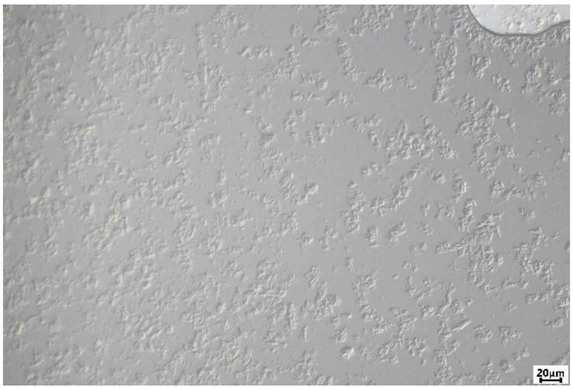

(**a**)

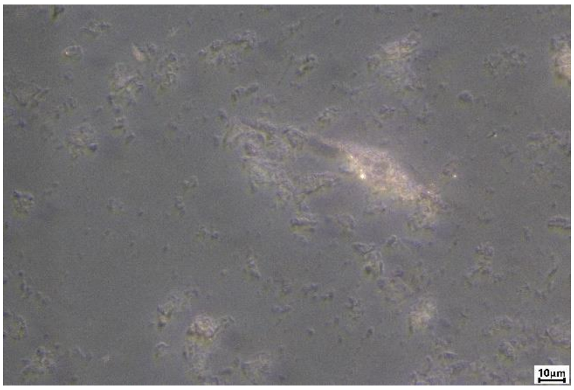

(**b**)

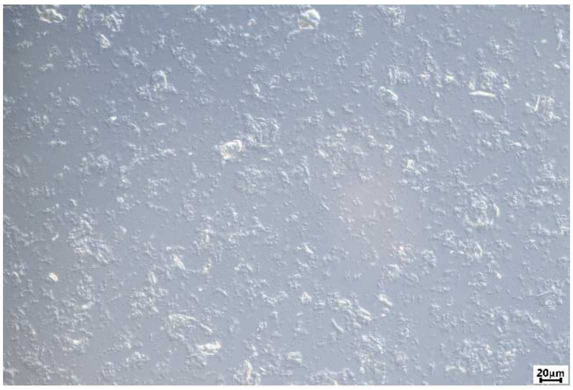

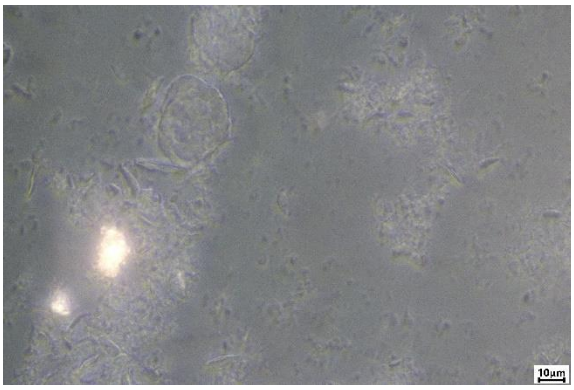

(c) **(d)**

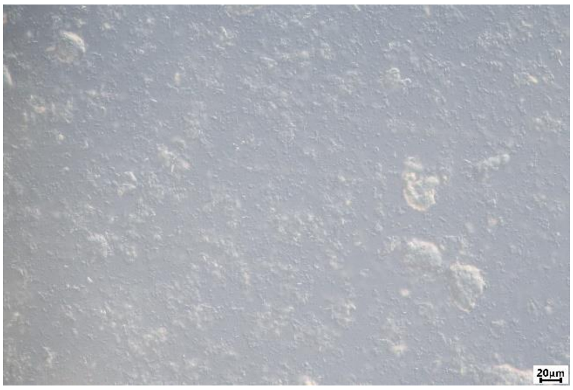

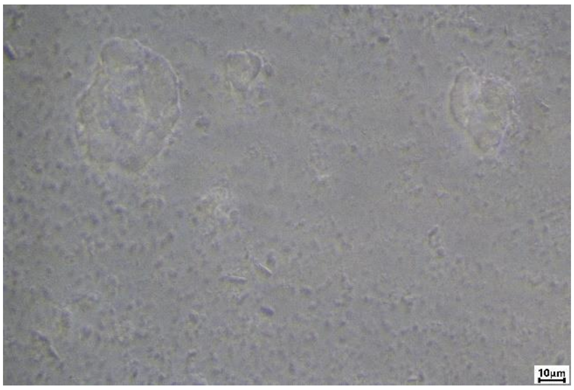

(e) **(f)**

Figure S2. Microphotography of suspension HA in mother solution: H_2O-3 (**a**), H_2O-7 (**b**), PVA2.5-3 (**c**), PVA2.5-7 (**d**), PVA5-3 (**e**), PVA5-7 (**f**)
